# “Walking a Day in My Shoes”: A Clinical Shadowing Program to Enhance Medical Students’ Understanding of Chronic Disease Management Beyond Clinical Settings

**DOI:** 10.3390/clinpract15050094

**Published:** 2025-05-13

**Authors:** Aidan Hilton, Waseem Jerjes

**Affiliations:** Department of Primary Care and Public Health, Faculty of Medicine, Imperial College London, London W12 0BZ, UK; aidan.hilton@nhs.net

**Keywords:** medical education, empathy, social determinants of health, experiential learning, patient-centred care

## Abstract

Aims/Background: Medical education is largely clinical and biomedical with little emphasis being put upon the social determinants of health (SDH) and patient-centredness. A programme entitled “Walking a Day in My Shoes” was devised as a pilot cohort study with the view of evaluating the impact of a clinical shadowing experience upon the empathy, SDH awareness, and patient-centredness of medical students. Methods: A prospective cohort study, involving 28 final-year London-area medical students, employing a three-phase teaching programme comprising preparation, observation, and reflection was carried out. Students’ confidence in the management of non-medical barriers, SDH awareness, and empathy before and after shadowing were measured using pre- and post-shadowing questionnaires. Qualitative analysis of patient feedback and journals also occurred. Results: Statistically significant improvements were observed in students’ empathy (mean score increase from 6.8 to 8.9, *p* < 0.001), understanding of SDH (advanced comprehension rose from 35% to 93%), confidence in addressing non-clinical barriers (from 39% to 86%), and awareness of logistical challenges (from 31% to 81%). Qualitative analysis highlighted key themes, including systemic barriers (transportation, polypharmacy, and social isolation) and students’ increased awareness of the emotional toll of chronic illness. Patients expressed high satisfaction, with 97% agreeing that the programme improved students’ understanding of chronic disease management. These findings suggest the programme’s practicality and scalability in medical education. Conclusions: This pilot cohort study demonstrated the significant enhancement of the students’ empathy, perception of SDH, and patient-centredness preparation through immersive shadowing. The findings support the use of experiential learning programmes as curricular interventions.

## 1. Introduction

As healthcare, and the expectations held by the patients it operates for, continues to evolve, greater emphasis is being placed on medical practitioners to be able to view patients more holistically and incorporate non-clinical factors into their decision-making processes. The social determinants of health (SDH) are the factors through which a person’s life, and the society they live in, affect their health [1]. Training future clinicians to have an awareness of the SDH has been identified both nationally and internationally as essential for undergraduate and postgraduate medical education [1,2,3,4].

Understanding SDH requires empathy, as clinicians must grasp how a patient’s health is shaped by broader life circumstances. Clinician empathy has been well established as a positive predictive factor for improving patients’ health, through areas such as improved medication adherence [5,6]. However, there is currently no consensus view on the best way to teach these principles to students, with methods ranging from communication skills training to ‘Balint’ group discussions [7,8,9]. While Balint groups and communication training provide structured reflection, they lack direct patient engagement, limiting students’ ability to appreciate real-world healthcare barriers.

Recent studies have explored multiple strategies for incorporating SDH into medical education, including checklists, structured case discussions, and direct community engagement, each with varying degrees of effectiveness in fostering long-term student engagement with social determinants [10,11,12].

Experiential learning, particularly through patient shadowing and direct engagement with social determinants, has been shown to enhance students’ ability to recognise and address systemic healthcare barriers, promoting more holistic and patient-centred clinical approaches [13,14].

By spending time with patients with common chronic diseases, such as diabetes, heart failure, or chronic obstructive pulmonary disorder (COPD), students can gain experience of how this cohort of patients struggle daily with problems that go far beyond clinical management. These include logistical difficulties such as coordinating complicated medication regimens, limited resources, or organising transport to healthcare settings, all whilst dealing with the often debilitating symptoms that necessitate these healthcare interventions in the first place. In addition to logistical difficulties, emotional and social burdens—such as isolation, stigma, and stress—further undermine patients’ health.

This study aims to bridge the gap through the evaluation of the effectiveness of a new shadowing programme with the aim of involving medical students in experiential chronic disease management.

By immersing them in the lives of patients through experiential learning, we believe practitioners in the future would be more sensitised about how social, emotional, and personal factors motivate patient behaviours and health outcomes beyond the traditional biomedical model.

This pilot study measures the benefit of experiential learning in enhancing the understanding of chronic disease management as well as social determinants of health (SDH) among medical students, ultimately enabling a more patient-centred model of healthcare. By bridging the divide between clinical training and the patient experience, the research aims to enable the enhancement of the procedures of medical education with emphasis on empathy, communication, and integrative care.

## 2. Materials and Methods

### 2.1. Study Design

This study applied a prospective cohort method to evaluate the effectiveness of an immersive clinical shadowing programme in enhancing final-year student awareness of chronic disease management, social determinants of health (SDH), and patient-centredness. Twenty-eight London-based medical students completed a three-phase programme: Preparation, Shadowing, and Reflection. During the preparation phase, students completed a pre-shadowing survey (Appendix A.1.1). Then, students completed two one-hour online workshops, held over two weeks, discussing the SDH, ethical engagement, and patient-centred care. The Shadowing phase had students spend one full day observing patients with chronic conditions in home and community environments. Finally, the Reflection phase included a structured, facilitated, debriefing session lasting one and a half hours, followed by students completing a post-shadowing survey (Appendix A.1.2) and then, submitting a reflective journal within one week (Appendix A.1.3). Students’ shifts in empathy, confidence in the management of non-clinical barriers, and awareness of systemic impediments were evaluated using pre- and post-shadowing questionnaires, with qualitative evidence from patient feedback and reflective journals supporting the evaluation.

### 2.2. Setting

This project was conducted in London medical schools and healthcare regions, with volunteer final-year medical students undergoing a shadowing scheme with the objective of enhancing their knowledge of chronic disease management and the social determinants of health (SDH). It was carried out during the period of [June 2023–June 2024], with student volunteers recruited via email invitations.

Students were paired with patients with chronic illnesses, like diabetes, chronic obstructive pulmonary disease (COPD), and cardiac disease. Pre- and post-shadowing questionnaires, qualitative analysis of reflection journals, and patient feedback served as measures of student changes in empathy, confidence in the management of non-medical barriers, as well as systemic awareness. Placement in the clinical area provided a realistic experience of patient management with exposure to the role of non-medical as well as clinical factors influencing the management of chronic disease.

### 2.3. Participants

Participants were recruited using convenience sampling. Twenty-eight invited students agreed to participate, and there were no dropouts or missing data for the pre- and post-questionnaires.

Students were eligible for inclusion if they were enrolled in the final year of their medical education and had completed the core clinical training in primary care, surgery, and medicine. All students were required to demonstrate basic knowledge of chronic disease management and SDH through pre-programme surveys and must have been willing to participate in all phases of the programme. Students who had prior experience in formal SDH training or had previously participated in similar shadowing programmes were excluded to minimise prior knowledge bias whilst ensuring the novelty of the intervention. Students who would be unable to participate in the whole programme, pre-shadowing workshops, or after-shadowing debriefing, or who had conflicting interests, such as pre-existing connections with the patients who would be participating, were excluded. Twenty-eight students gave written consent to participate.

Patients with chronic illnesses were recruited from community and general practice settings to ensure diverse representation. Patients with a confirmed diagnosis of at least one chronic condition—for instance, chronic obstructive pulmonary disease (COPD), diabetes, or heart failure—who were self-managing their condition with active contact with healthcare providers and healthcare services were considered. Patients required agreement to participate in a day-long shadowing experience, wherein the student would accompany them and shadow their daily health management concerns, as well as written consent after being fully aware of the aim and scope of the study. For diversity, patients from varied age brackets, socioeconomic statuses, and healthcare settings (e.g., home-based healthcare, outpatient departments, and primary healthcare visits) were recruited. Patients with advanced cognitive impairment or illness affecting their ability to provide informed consent, for instance, cognitive impairment, as well as patients requiring extensive medical interventions for the duration of the study, for instance, hospitalisation or chronic illness worsening, were excluded. Patients who would be uneasy with student shadowing or have privacy concerns about their home or healthcare interaction were also excluded. Fifteen patients gave written consent to participate

Students were matched at random with patients to minimise selection bias as well as optimise exposure to diverse patient experiences. Matching occurred with no pre-existing doctor–patient relationship being present to prevent bias. Logistical feasibility, as well as geographic convenience, was also considered as the process proceeded. Patients were matched with students based on availability rather than disease type to avoid bias, ensuring exposure to a range of chronic illness experiences.

### 2.4. Variables

The primary interventions in this study were the level of the students’ empathy, social determinants of health (SDH) awareness, and confidence in the management of non-clinical patient-care hindrances. Students rated their level of empathy using a 10-point Likert scale pre- and post-shadowing. SDH awareness was assessed using categorical answers, with the students rating their level of awareness as being elementary, moderate, or advanced/extensive. Students rated agreement with statements about confidence in the management of non-clinical patient-care hindrances using a 5-point Likert scale.

Secondary outcome measures included awareness of systemic and logistic barriers, as evidenced through student reflection journal responses as well as qualitative responses from the post-shadowing debrief. Thematic qualitative analysis was applied in the examination of the presence of specific themes such as transportation difficulties, compliance with medications, and social isolation. Patient opinions were also gathered as an exploration measure with feedback about their level of comfort with student shadowing, their perception of student interaction, as well as their views about the value of the programme in influencing the next generation of physicians.

Potential confounding variables included exposure to chronic disease management prior to the study, experience with patient interaction outside the clinical setting, and their clinical placement at the study point (medicine, primary care, or surgery). These variables were ascertained from the pre-shadowing questionnaire and controlled for through the analysis given the pre-existing variance among the participants.

### 2.5. Data Sources and Measurement

Data were collected through a combination of quantitative questionnaires, qualitative reflection diaries, and patient feedback questionnaires. Pre- and post-shadowing questionnaires, designed by the authors, were completed by students to assess change in empathy, understanding of SDH, and confidence in managing non-clinical barriers. The content of the questionnaires was based on established frameworks of medical education and SDH awareness. Empathy was quantified on a 10-point Likert scale, while SDH awareness and confidence were assessed using categorical and 5-point Likert scale responses. Questionnaires aimed to assess self-perceived changes among the students and were completed just prior to and following the shadowing programme.

The empathy-related items were adapted from the Jefferson Scale of Empathy (JSE) for reflective use after experiential learning. Content validity was confirmed through expert review by a senior medical educator. As the post-experience questionnaire was context-specific and used in a single administration, internal consistency measures (e.g., Cronbach’s alpha) and test–retest reliability were not assessed.

The questionnaire underwent an expert review process, including input from clinical educators, to ensure content validity. A pilot version of the questionnaire was tested with a small group of students, yielding a reliability index of 0.85, demonstrating strong internal consistency.

Reflective journals were the method used in recording observations, insights, as well as the meaningful learning experience following the process of shadowing. Students were given structured prompts, such as the following:

- “Describe how the patient’s environment and social circumstances affected their health management”.
- “What were the most unexpected challenges the patient faced in navigating the healthcare system”?
- “Reflect on how this experience has changed your perspective on patient-centred care”.

Thematic analysis was utilised in the process of finding recurring patterns relating to systemic impediments, logistic challenges, and emotional reflections. To ensure depth and authenticity, reflections were evaluated based on thematic richness rather than general descriptive accounts. Qualitative data from reflective journals and patient feedback were analysed using Braun and Clarke’s six-step framework, which involves familiarisation with data, coding, generating themes, reviewing themes, defining themes, and producing the final report.

Patient feedback questionnaires were used to measure the experience from the patient’s perspective, their comfort with student shadowing, perceptions of interaction with the student, and views regarding the value of the programme in influencing the student’s clinical practice in the future. Responses included measures using the Likert scale as well as free-text comment, which were coded for the presence of recurring themes regarding the value and acceptability of the experience. To ensure confidentiality, patient feedback was anonymised at the point of collection, with no identifiable information recorded or stored.

All data were self-reported and no clinical records were accessed. Questionnaires and journals were anonymised before analysis to preserve confidentiality. Data collection procedures were identical for all participants in order to preserve comparability, and no patients or students were excluded on the grounds of incomplete responses.

### 2.6. Intervention Structure

The study used a three-phase approach—Preparation, Shadowing, and Reflection—designed to guide students through a structured learning process focusing on real-world patient experiences and systemic healthcare challenges (Table 1).

#### 2.6.1. Preparation Phase

Students completed a pre-shadowing survey (Appendix A.1.1). Students participated in an interactive online workshop covering SDH, patient-centred care, and ethical engagement. The case studies highlighted systemic barriers, such as a patient missing an appointment due to unreliable transportation. These discussions encouraged students to critically analyse how systemic challenges affect health outcomes. Ethical training emphasised confidentiality, respecting boundaries, and understanding the students’ non-intrusive observer roles.

The interactive online workshops were conducted over a period of two weeks, with two 1 h sessions per week. Each session was designed to cover different aspects of social determinants of health and patient-centred care. The workshops were hosted on the Zoom platform, which allowed for live discussions, real-time Q&A, and engagement with relevant multimedia materials to enhance learning.

Students were paired with volunteer patients managing chronic conditions, such as diabetes, COPD, or heart failure. The pairing ensured exposure to diverse socio-demographic backgrounds and healthcare settings, preparing students for the complexities of shadowing. Pre-shadowing surveys further assessed their initial perspectives, confidence in understanding patients’ logistical and emotional needs, and learning expectations.

#### 2.6.2. Shadowing Phase

Students spent a full day observing their assigned patients’ daily routines and healthcare interactions. Activities included documenting medication management, dietary restrictions, and other daily challenges. Students accompanied patients to GP visits, outpatient clinics, or community care settings to gain insight into logistical and emotional barriers in accessing care. They observed interactions with healthcare providers like doctors, pharmacists, nurses, and administrative staff, identifying systemic inefficiencies such as transportation issues or inadequate access to resources. Non-intrusive observation, as described in patient-centred experiential learning models, ensures that students passively observe without interfering with patient interactions, allowing for an authentic understanding of healthcare experiences while minimising potential behavioural modifications.

Post-shadowing surveys measured changes in empathy, understanding, and confidence. Students reflected on statements like, “This experience helped me understand the non-clinical challenges patients face in managing their health”, and described their most valuable lessons.

#### 2.6.3. Reflection Phase

Students participated in group debriefing sessions led by one clinical educator (author WJ) to share observations and discuss solutions to systemic barriers. They synthesised their learning by completing reflective journals addressing prompts like, “Describe how the patient’s environment and social circumstances affected their health management”, and “What surprised you most about the patient’s healthcare experience outside the clinical setting”?

Patients provided feedback through post-shadowing surveys (Appendix A.1.2), rating their comfort with having a student shadow them, respect for privacy, and the programme’s potential to improve medical education. Students, further, submitted a reflective journal within one week (Appendix A.1.3). Open-ended responses allowed patients to share additional insights and suggestions for improvement (Appendix A.2).

Although the debriefing sessions were guided by a single facilitator (author WJ), efforts were made to minimise facilitator bias and standardise the learning environment across students. The facilitator followed a semi-structured discussion guide to ensure that all key themes were addressed consistently. To reduce potential variability, facilitator input was limited to prompting discussion and encouraging balanced participation, rather than introducing new interpretations or themes. No students were assessed or graded by the facilitator, which helped foster an open and non-hierarchical space for reflection. Furthermore, the students’ reflective journals—written independently post-debrief—provided an additional source of data for triangulation, allowing the researchers to confirm whether facilitator presence had influenced student responses. This layered approach helped preserve the reliability and objectivity of the qualitative findings.

### 2.7. Bias

Several steps have been undertaken to minimise bias in this research. Selection bias was limited through the invitation of a heterogeneous set of final-year clinical student volunteers from multiple clinical rotations/medical schools with random allocation of patients to students to prevent preferential allocation. Response bias was limited through the assurance of participants that responses from the questionnaire and reflection diaries would be anonymised, facilitating honest feedback. Observer bias was limited through the pre-briefing of the role of the student as non-intrusive observers through the process of shadowing with pre-shadowing training in the use of objective observing as well as ethical participation. Patient feedback social desirability bias was limited through the collection of patient feedback forms being completed confidentially without any interaction with the researchers. The lack of control group as well as the use of self-reported measures were limitations since the participants could exaggerate the gain in their learning.

To minimise potential subjective bias, debriefing sessions were structured to allow for input from all students, with a focus on open-ended questions that encouraged diverse perspectives.

### 2.8. Study Size

The sample size was determined based on the estimated effect size required to detect meaningful improvements in students’ empathy, understanding of social determinants of health (SDH), and confidence in addressing non-clinical barriers. Using a paired *t*-test design with an expected mean change of 1.5 points (SD = 2.0) on a 10-point Likert scale for empathy scores, a power of 80% (β = 0.20), and a two-tailed α of 0.05, the minimum required sample size was calculated as 24 students. To account for potential dropout and incomplete data, the recruitment target was set at 28 students, ensuring adequate statistical power to detect significant pre- and post-intervention differences. Given the exploratory nature of this pilot cohort study, this sample size was deemed appropriate for generating preliminary evidence while allowing for future replication with larger cohorts.

### 2.9. Quantitative Variables

The primary quantitative measures collected in this research comprised empathy, SDH understanding, and confidence in the management of non-clinical barriers. A 10-point Likert scale was used to measure empathy, with higher levels representing higher self-assessed empathy. SDH understanding was classified as basic, moderate, or advanced/comprehensive, with pre- and post-shadowing changes evaluated for enhancement. A 5-point Likert scale from strongly disagree (1) through strongly agree (5) was utilised to measure confidence in the management of non-clinical barriers like transportation difficulties and medication compliance. Other quantitative measures comprised awareness of logistic barriers among the students, pre- and post-programme changes in the recognition of systemic healthcare difficulties, as well as patients’ satisfaction levels with the experience of being shadowed. Continuous variables like the score of empathy were compared using paired *t*-tests, whereas categorical variables like SDH comprehension as well as levels of confidence were compared using chi-square tests for changes in proportions. Quantitative measures facilitated the objective determination of the programme’s impact as well as the provision of statistical validity for the qualitative results.

### 2.10. Statistical Methods

Descriptive and inferential statistical analyses were conducted to assess the impact of the shadowing programme on students’ empathy, understanding of SDH, and confidence in addressing non-clinical barriers. Continuous variables, such as empathy scores, were presented as means and standard deviations (SDs), while categorical variables, including SDH comprehension levels and confidence in addressing non-clinical barriers, were summarised as frequencies and percentages. Pre- and post-shadowing comparisons of continuous variables were analysed using paired *t*-tests, with a significance level of *p* < 0.05 considered statistically significant.

Likert-scale items such as empathy scores were aggregated and treated as continuous variables. The distribution of these summed scores was tested using the Shapiro–Wilk test, which confirmed normality assumptions were met. As a result, a paired *t*-test was applied to assess differences in means before and after the intervention. Chi-square tests were used for categorical comparisons (e.g., proportions of students agreeing with specific statements). These methods were selected to appropriately handle both continuous and categorical pre- and post-intervention data.

Categorical data were analysed using chi-square tests to evaluate proportional changes in students’ self-reported understanding and confidence levels. Effect sizes were calculated using Cohen’s d, where values of 0.2, 0.5, and 0.8 corresponded to small, medium, and large effects, respectively. The normality of data distribution was assessed using the Shapiro–Wilk test, and assumptions for the paired *t*-test were met.

To assess potential confounding variables, subgroup analyses were conducted based on students’ clinical rotation at the time of participation (primary care, medicine, or surgery). A one-way ANOVA test was used to determine whether baseline differences in empathy scores existed between subgroups. Additionally, a linear regression model was applied to explore whether students’ prior exposure to chronic disease management predicted the magnitude of change in empathy and SDH comprehension. Sensitivity analyses were conducted to evaluate the robustness of findings, including testing for outliers and checking assumptions for normality.

Missing data were minimal, and a complete case analysis approach was used given the small sample size. No adjustments for multiple comparisons were made due to the exploratory nature of this pilot study.

### 2.11. Ethical Considerations

This study was reviewed using the UK Health Research Authority (HRA) decision tool, which confirmed that NHS Research Ethics Committee (REC) review was not required, as the project did not involve NHS services or the use of identifiable patient data. All participants provided informed consent, and the study was conducted in accordance with institutional ethical guidelines for educational research. A copy of the HRA decision outcome is included as a Supplementary Materials.

Participation in the research was voluntary, with all the students having consented in writing before being accepted onto the programme. Patients who gave consent to being shadowed also consented after being fully briefed regarding the objectives of the research, the procedures, as well as the non-intrusive role of the student. For maintaining confidentiality, all the data were anonymised, with the participants being assigned unique study identifiers with no personally identifiable information being gathered. Responses from the surveys as well as the reflection journals were stored as per the policy of data protection.

This study was conducted in accordance with the Declaration of Helsinki, ensuring that all participants were fully informed about the nature of the study, their voluntary participation, and their right to withdraw at any time without consequence.

### 2.12. Figure Design Software

The software used for figure design was IBM SPSS Statistics, Version 30.0.0, which was released in September 2024 by IBM Corporation, Armonk, NY, USA.

## 3. Results

### 3.1. Participants

**Figure 1:**
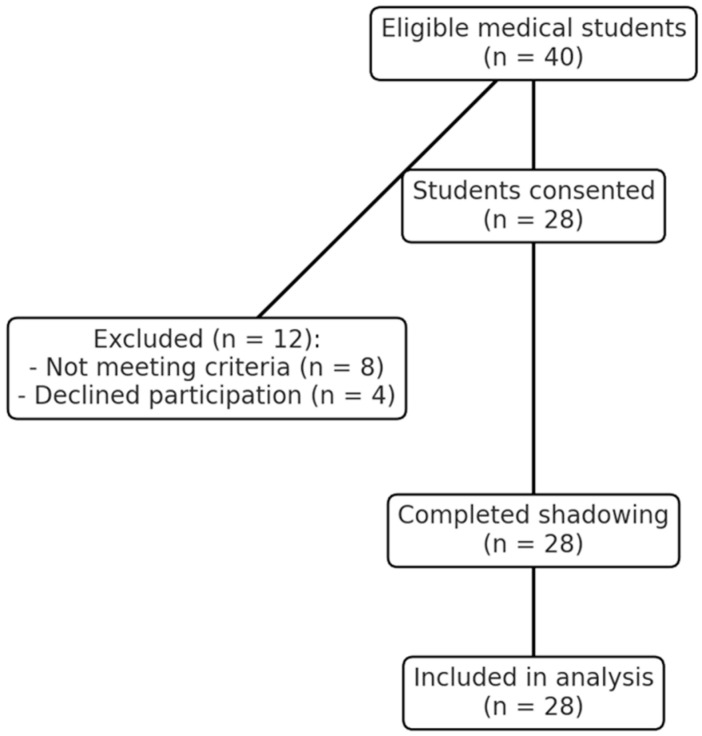
Flow diagram of participant recruitment and inclusion.

A total of 40 final-year medical students from medical schools in the London area were invited to participate. Of these, 28 students (70%) consented and met the inclusion criteria, comprising 16 females (57%) and 12 males (43%), with a mean age of 24.5 years (range: 23–26 years). The remaining 12 students were excluded, including 8 who did not meet eligibility criteria (e.g., prior formal patient shadowing experience beyond standard medical training) and 4 who declined participation due to time constraints or other commitments (Figure 1).

All 28 enrolled students successfully completed the full three-phase programme: Preparation (online workshops on SDH and ethical engagement), Shadowing (full-day patient observation), and Reflection (structured debriefing and journaling). No students withdrew or were lost to follow-up.

The 15 patients who participated in the study had a mean age of 67.3 years (range: 52–81 years), with 9 (60%) being female and 6 (40%) male. The majority (73%) were retired, with varied socioeconomic backgrounds, including low-income (27%), middle-income (47%), and higher-income (26%) groups. Chronic conditions represented included diabetes (40%), COPD (33%), and heart failure (27%). Patients were recruited from local healthcare settings, including general practice and community healthcare services, and paired with students based on availability and clinical relevance.

Each student shadowed one patient for the full day, ensuring exposure to diverse healthcare settings (home-based care, outpatient clinics, and primary care visits). No participants withdrew from the study.

### 3.2. Impact on Students

**Figure 2:**
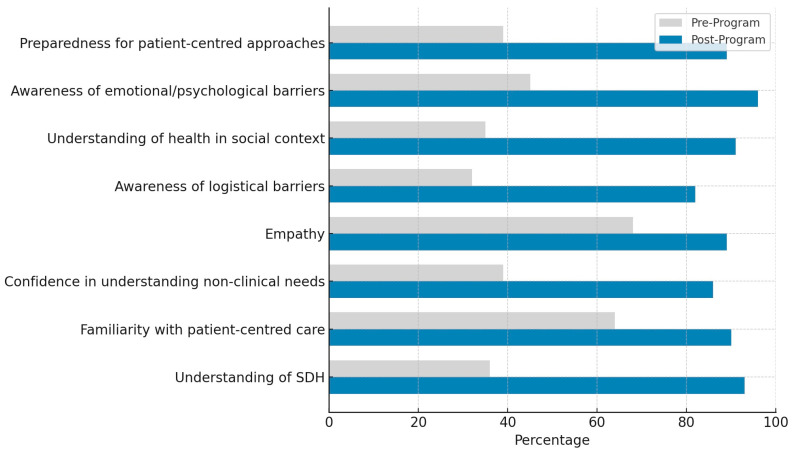
The pre- and post-programme outcomes across eight learning domains. Each bar represents the percentage of students reporting agreement, awareness, or advanced understanding in the specified domain before and after participating in the shadowing programme.

The shadowing programme profoundly influenced students, as shown through pre- and post-shadowing surveys, reflective journals, and debriefing sessions. Students demonstrated statistically significant changes in their understanding of the social determinants of health, empathy, and confidence in delivering patient-centred care, as measured by pre- and post-programme assessments (Figure 2).

Before the programme, students showed limited knowledge of social determinants of health (Table 2). Only 36% agreed or strongly agreed that they understood how factors such as transportation, housing, and income affect patient health, with 40% remaining neutral. While 64% of students were familiar with the concept of patient-centred care, just 39% felt confident addressing non-clinical barriers like logistical or emotional challenges. On a 10-point scale, empathy was rated at an average of 6.8 ± 1.2, with only 45% describing their empathy as “high”. Despite these gaps, 86% of students strongly agreed they hoped to learn more about the challenges faced by patients with chronic conditions.

Following the shadowing experience, students reported significant gains across all evaluated domains. Understanding of social determinants of health rose dramatically, with 93% of students rating their knowledge as “advanced” or “comprehensive”, compared to just 36% pre-programme (*p* < 0.001). Agreement with the statement, “I now have a better understanding of how social factors (e.g., income, education, family) impact patient health” increased from 35% to 91%. Male students showed the largest improvement, with comprehension rising from 28% to 93%, compared to an increase from 59% to 91% among female students.

Empathy scores rose significantly to 8.9 ± 1.1 (*p* < 0.001), with 96% of students agreeing that the experience enhanced their empathy. Primary care students showed the largest empathy gains, increasing from a pre-programme mean of 6.38 (±0.23) to a post-programme mean of 8.88 (±0.23), reflecting a mean change of +2.5 points. Surgery students followed, with empathy scores rising from 6.46 (±0.22) to 8.56 (±0.22), a mean change of +2.1 points. Medicine students exhibited the smallest increase, with scores improving from 6.56 (±0.22) to 8.36 (±0.22), a mean change of +1.9 points. One-way ANOVA confirmed that these differences were statistically significant (*p* < 0.05). Confidence in delivering patient-centred care also rose substantially, with 86% agreeing or strongly agreeing post-programme, compared to 39% pre-programme (*p* < 0.01). Awareness of logistical barriers, such as transportation and scheduling, increased from 32% to 82% (*p* < 0.01). Moreover, 89% of students stated that the experience would influence their future clinical approach, compared to 41% before the programme (*p* < 0.01).

A paired *t*-test demonstrated a significant increase in empathy scores (*p* < 0.001), with a large effect size (Cohen’s d = 1.82; 95% CI: 1.31–2.33), indicating a substantial improvement post-programme. A chi-square test confirmed this increase was statistically significant (χ^2^(1) = 12.65, *p* < 0.001), with a moderate-to-large effect size (η^2^ = 0.23), suggesting a meaningful shift in students’ confidence.

**Figure 3:**
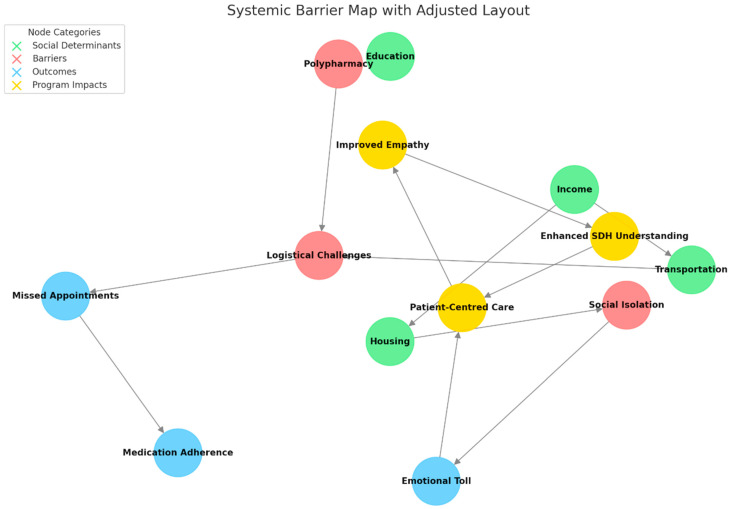
Systemic barrier map with intervention points. This figure illustrates the interconnected relationships between social determinants of health, systemic barriers, and health outcomes. It highlights critical intervention points, such as addressing transportation and polypharmacy, that can improve patient-centred care. The visualisation underscores the importance of integrating non-clinical factors into healthcare decision-making.

Reflective journals added depth to these findings (Table 3). Students described observing systemic barriers like unreliable transportation, social isolation, and challenges with polypharmacy (Figure 3). These observations helped them understand the complexities of managing chronic disease. Many expressed surprise at patients’ resilience and recognised the emotional toll of chronic illness. One student wrote, “Seeing the patient’s daily struggles made me realise how much happens outside the clinic that we often overlook”.

Students also proposed actionable strategies for improving care delivery, including expanding home-based care, introducing patient navigators, and enhancing provider training in empathy and patient-centred communication. These reflections showed a shift in clinical perspective, with many highlighting the importance of addressing non-clinical factors and adopting a more holistic approach. As one student summarised, “This programme taught me to see patients as individuals with unique challenges, not just cases or symptoms”.

### 3.3. Impact on Patients

Patients gave overwhelmingly positive feedback, highlighting the programme’s reciprocal value (Table 4). Ninety-six percent agreed or strongly agreed they felt comfortable having a student shadow them, and 93% reported that students respected their privacy and personal space. Additionally, 100% believed the programme could improve how future doctors understand and treat chronic illnesses. One patient noted, “The student genuinely wanted to understand what it’s like to live with my condition. It was refreshing to feel heard”.

Patients emphasised the programme’s value in educating future healthcare providers, noting it allowed students to see the realities of managing chronic illness, particularly non-clinical challenges often overlooked in clinical settings. Ninety-one percent of patients said they would recommend the programme to others. However, a few suggested students spend more time asking open-ended questions to better understand patients’ perspectives on healthcare.

Open-ended responses revealed further insights. Patients appreciated the students’ respectful approach, with one commenting, “The student listened more than they spoke, which made me feel comfortable sharing my experiences”. Another patient noted, “Doctors need to see the daily challenges their patients face, not just the symptoms presented in the clinic”. These responses illustrate that the programme not only enhanced students’ learning but also gave patients a sense of contributing to medical education improvement.

Several students reflected on how the experience would influence their future patient interactions. One participant noted, “I’ll think more about what happens after the appointment ends—how patients actually cope at home.” Others emphasised how seeing patients’ homes gave them context for future clinical decisions. These reflections suggest that shadowing may enhance not only empathy but also practical clinical judgement.

## 4. Discussion

### 4.1. Key Results

The “Walking a Day in My Shoes” shadowing experience enhanced the awareness of SDH, empathy, as well as confidence in the management of non-clinical barriers among the student doctors. A mere 36% of the students rated their SDH awareness as extensive or advanced before the programme, but this improved to 93% after the programme, indicating significant awareness of systemic healthcare challenges. Similarly, the level of empathy improved from 6.8 pre-programme to 8.9 post-programme on a 10-point scale, indicating the higher emotional connection with patient experience.

In addition to heightened awareness and compassion, there also came a notable increase in confidence in the power of approaching healthcare impediments, from 39% pre-programme to 86% post programme. Observing patients struggle with the logistic complexities, social isolation, and the complexities of polypharmacy provided the students with concrete exposure to the complexities of chronic disease management, affirming the value of patient-centredness.

Patient feedback was overwhelmingly positive, with all participants agreeing the programme would help future doctors better understand chronic disease management. Patients valued the consideration afforded to their experience and the opportunity to contribute to the teaching of medicine. These results illustrate the twofold value of experiential learning, with patients as much as the students benefitting from each other.

Emerging themes from students’ reflections included an increased empathy for patients’ non-clinical challenges, while patient feedback highlighted systemic barriers such as inadequate healthcare access and transportation issues, with these themes significantly influencing students’ understanding of comprehensive patient care.

### 4.2. Limitations

This study is constrained in a variety of ways, the majority of which can be applied more generally in the research of medicine. A limitation is the low number of 28 participants, since more would have greater statistical power as well as a more heterogeneous measure of the programme’s impact. While effect sizes indicate meaningful changes, a larger sample is necessary to validate these results across diverse student populations. Future studies should aim for larger, multi-institutional cohorts to enhance statistical power and generalisability; recruiting a larger and more diverse student cohort from multiple institutions and regions would ensure that the observed effects are not setting-specific and could be broadly applied to different healthcare education environments.

A further limitation is the risk of pre- and post-study questionnaire response bias. Students may have over-reported gains in empathy or SDH awareness due to perceived expectations. Although the students were assured honest feedback would be utilised for programme enhancement, this sort of bias is always difficult to remove entirely despite reassurances that feedback would be anonymous. Future studies should consider blinded assessments or objective measures of empathy to mitigate this limitation.

Similarly, while 100% of (the 15) patients reported that the programme would help future doctors understand chronic illness better, this high approval rating may be influenced by social desirability bias, as patients may have felt inclined to provide positive feedback due to their direct interaction with students.

The effect size of the empathy also raises questions about clinical significance. As much as there is evidence from the research supporting the link between patient outcomes and clinician empathy, the question is how much extra empathy is there before there is no clinical value added. Objective measures of patient outcomes were absent from the study, prohibiting the determination of how the higher levels of SDH awareness and enhanced levels of empathy would be correlated with significant patient care improvements. Long-term evaluations could include patient feedback on clinician empathy, structured assessments of students’ SDH-related clinical decision-making, and healthcare quality indicators such as adherence rates and patient engagement.

Finally, the brevity of the study precludes measurement of long-term retention of SDH awareness and compassion. Students’ perceptions were measured just before the programme as well as after the programme, but no one can be sure the changes endure. To determine the sustainability of the observed improvements in empathy, SDH understanding, and confidence, future studies should incorporate a longitudinal design, assessing students’ clinical practice and patient interactions six months to several years post-programme, to assess how long the experiential experience continues to impact clinical practice as well as attitudes toward patient care.

### 4.3. Interpretation

Prior studies by Balhara et al. and Nguyen et al. demonstrated that engagement with the community by experiential learning was beneficial to teaching the SDH; however, these studies were limited to observing the entire community as opposed to the more direct one–one approach we have suggested here [10,14].

Our findings support shadowing as an effective method for teaching SDH, empathy, and non-clinical care skills. Thematic analysis allowed for a deeper understanding of how experiential learning shifted students’ views on patient-centred care, moving beyond clinical knowledge.

Significant improvements in all these areas indicate that biomedical teaching alone is possibly ineffective in preparing the next generation of clinicians with the skills necessary to deal with the complexities of chronic disease management. By exposing students to real patient experiences, the programme bridged the gap between theory and patient-centred practice. Empathy improvements have been linked to enhanced patient trust and adherence to treatment plans, while greater confidence in addressing non-clinical barriers equips future clinicians with the skills needed to navigate complex patient care environments, potentially improving long-term health outcomes.

Our study supports the growing body of literature that highlights the importance of integrating experiential learning in medical education to enhance empathy and the understanding of SDH [10,13,14,15]. Our research found that students demonstrated significant improvements in empathy, particularly during primary care rotations whereas internal medicine rotations resulted in smaller empathy gains compared to primary care and surgery. This discrepancy may be explained by the focus of medicine rotations on acute disease management, which may not provide as much direct exposure to patients’ emotional and social challenges.

These findings underscore the value of primary care in medical education, where students are exposed to patients’ long-term health conditions and the broader social context of their care. In contrast, rotations in other specialties may require more focused strategies to ensure that students develop a similar depth of understanding of SDH and empathy.

While the immediate benefit in terms of empathy and SDH awareness is seen, the research does not ascertain the long-term retention of these changes or the actual impact on clinical decision-making. Future research would be necessary to see if the students who undergo these kinds of programmes continue with their enhanced awareness and apply more patient-centred models of care in their later work. Future studies could implement a longitudinal follow-up design where students are reassessed six months to one year post-programme to evaluate the retention of empathy and SDH awareness. This could include real-world application measures, such as patient feedback on student–clinician interactions.

Further, the largely positive patient feedback also points the way toward the dual benefit of these kinds of programmes: improving student education as well as patients feeling listened to and considered as part of the healthcare system. These results contribute to the body of evidence for greater experiential learning within the medical curricula. The findings affirm the imperative of reform in medical teaching with priority to social determinants, patient experience, and interdisciplinary models, in order to better prepare physicians for the complexities of chronic disease management.

### 4.4. Generalisability

The findings of the research conducted here have limited generalisability since the programme is being implemented with a limited number of students from the London area. Findings may differ in other healthcare environments, institutions, or health systems. A more extensive programme with multiple hospitals and medical schools, as well as multiple networks of primary care, would present a more realistic picture of the programme’s impact.

Additionally, the study also targeted final-year medical students who would have possibly gained higher levels of clinical awareness as well as empathy compared with earlier-year students. programme effectiveness can also be varied with trainees at different levels of their studentship, and the programme’s impact should be explored with more cohorts, including pre-clinical students as well as postgraduate trainees.

Cultural and healthcare system differences must also be considered in extrapolating these findings to other countries. Systematic barriers, SDH, and patient access to healthcare may be very disparate from healthcare system to healthcare system, potentially influencing the validity and usefulness of the shadowing experience. Application in rural, underserved, or international settings would also provide information on the programme’s scalability and feasibility outside our current model. In underserved or rural areas, where limited healthcare access and transportation barriers are common, the programme could be adapted by offering virtual shadowing experiences or coordinating transportation support for patients. Additionally, cultural norms related to healthcare may vary; therefore, including cultural competence training for students could help them better understand and engage with patients from diverse backgrounds.

Despite these limitations, the vast majority of student as well as patient feedback demonstrate the extensive educative value of experiential learning through shadowing. Increased use of the same model programme can be invaluable in the advancement of medical education, the creation of empathy, and the enhancement of patient-centred awareness in diverse clinical environments. Unlike traditional lecture theatre teaching, which primarily focuses on theoretical knowledge, and standard clinical internships, which emphasise clinical decision-making within structured environments, experiential learning immerses students in the lived realities of patients, allowing them to witness first-hand how social determinants impact health and healthcare interactions.

Future work could explore this model in a wider range of populations and settings, including rural or international contexts, to assess how cultural, systemic, or geographic differences influence both student learning and patient engagement. Expanding the scope of the programme would allow evaluation of its adaptability and broader impact.

As previously highlighted, the small sample size of 28 students, while appropriate for a pilot, limits generalisability. Adaptation of this shadowing model in other environments—such as rural settings, different health systems, or international medical schools—would allow exploration of contextual factors that may shape both student learning and patient experience. Pilot testing across varied institutional settings could help refine a flexible implementation framework.

### 4.5. Challenges and Future Directions

The “Walking a Day in My Shoes” programme revealed the systemic challenges patients with chronic conditions experience as well as the frustrations the medical students experience when implementing the process of shadowing. A prominent issue revealed was the experience of logistic challenges, such as transportation issues. Most patients revealed the process of even travelling for their appointments as overwhelming, with the factors of long distances, limited modes of transportation as well as the inability to afford the same being the reason for delayed or cancelled treatments. These findings affirm the requirement of healthcare systems being patient logistic conditions accommodating, more specifically for patients with chronic conditions. Addressing the disconnect between medical and social services requires integrating SDH-focused training into medical curricula and fostering interdisciplinary case management approaches. Policy-level solutions, such as increased funding for patient navigation programmes, may also help bridge these systemic gaps.

Another recurring issue was social isolation, notably in patients who live alone. A number of participants described the emotional burden of chronic disease, with the added complications of limited mobility and social isolation. Students witnessed with their own eyes how the social factors contribute toward patient health, highlighting the need for more interdisciplinary work involving clinical as well as social determinants of health.

From a clinical perspective, the control of polypharmacy was found to be a considerable problem. Patients with multiple long-term illnesses routinely struggled with intricate drug regimens, with ensuing bewilderment, non-compliance, and erratic supply from the pharmacy. Students took the cognitive and organisational burden patients have when managing their treatments, underpinning the importance of effective counselling with medicines as well as the role of the pharmacist in chronic disease management. For students, the biggest eye-opener was the emotional strain of seeing patients’ day-to-day struggles. Most reflected upon how their perception of healthcare changed, as they gained the realisation of the intricacies of living with chronic disease. This would suggest experiential learning is highly valuable but also there is value in having formal debrief with clinical educators as a process of processing the experience, gaining emotional resilience, and talking about ethical issues.

Moving forward, the programme can be extended to interdisciplinary training with the inclusion of pharmacy, social work, and nursing students with the aim of developing cooperative learning as well as improved collaboration among healthcare professional fields. Expanding the programme to include other healthcare professionals could enhance team-based learning; nursing students could offer insights into bedside care and patient advocacy, pharmacy students could highlight medication management challenges, and social work students could provide expertise on navigating healthcare access and social support systems, creating a holistic learning experience. Joint activities, such as interdisciplinary debriefings, case-based discussions, and collaborative patient-care planning exercises, would provide students with a broader understanding of healthcare roles and encourage a team-based approach to addressing SDH.

To ensure the programme is inclusive, partnerships with local community organisations could facilitate student learning about the specific social determinants affecting the population, such as employment, education, and housing. Tailoring the programme to include community health worker involvement could bridge gaps in care and provide students with a more holistic view of patient needs. While the immediate post-programme improvements in empathy and SDH awareness were encouraging, their long-term durability remains unknown.

Longitudinal data were not collected, and we are therefore unable to assess the sustainability of the improvements in empathy and SDH awareness. Future studies should incorporate delayed follow-up to examine whether attitudinal and behavioural changes are retained over time.

Longitudinal shadowing, with the same patient being accompanied by the same student for the long term, can give the student a more realistic view of the long-term trajectory of chronic disease.

A further limitation of the study is the lack of longitudinal follow-up. While immediate improvements in empathy and awareness of social determinants of health were evident, it remains unclear whether these effects are sustained over time. Future studies should explore the durability of these changes through delayed post-intervention assessments.

These findings suggest that incorporating patient-led, home-based experiences into medical education could lead to more personalised, context-aware care. By understanding patients’ daily routines, students may become more attuned to barriers such as mobility, literacy, and social support that affect adherence and outcomes.

## 5. Conclusions

The “Walking a Day in My Shoes” programme proved highly effective in teaching empathy and social determinants of health. By immersing the student into real-life patient circumstances, the programme has been identified as a very effective way of experiential learning in learning key skills required for patient-centred care. These findings add to the growing evidence base for the need for empathy and social awareness as key competencies for the future generation of physicians in the delivery of effective healthcare.

Additional programme growth, as well as a longitudinal study design, would establish its long-term impact on clinical empathy and patient-centred care. Moreover, adapting this approach to other healthcare students may promote interprofessional collaboration and enhance the quality of healthcare education overall.

Future work will focus on adapting and piloting the programme in multiple medical schools and healthcare settings, supported by co-design with faculty and patients. Developing facilitator training modules and implementation toolkits will be key to enabling wider adoption. 

## Figures and Tables

**Table 1:** The structured phases of the “Walking a Day in My Shoes” shadowing programme (an overview of the key activities, learning objectives, and skills developed).

Phase	Key Activities	Learning Objectives	Skills Developed
Preparation	- Pre-shadowing workshop on social determinants of health, patient-centred care, and ethical considerations. - Introduction to real-life challenges patients face (e.g., social, economic, logistical). - Case studies of patients with chronic illnesses to prepare students for diverse patient backgrounds. - Pairing of students with volunteer patients who are managing chronic conditions in various care settings.	- Understand the role of social, economic, and environmental factors in health outcomes. - Develop an empathetic approach towards non-clinical barriers affecting patient care. - Foster a patient-centric perspective before shadowing.	- Ethical sensitivity and confidentiality awareness. - Understanding of social determinants of health. - Preparation for active observation and non-intrusive participation.
Shadowing	- Full-day immersion with assigned patients in their home, community, or outpatient settings. - Observation of how patients manage health (medications, dietary restrictions, physiotherapy exercises). - Accompanying patients to GP or outpatient appointments, physiotherapy, or routine check-ups. - Observing interactions with healthcare providers, community nurses, pharmacists, and support services. - Witnessing daily life challenges like social isolation, limited mobility, or navigating public transportation to appointments.	- Gain real-world experience of how chronic conditions are managed outside clinical settings. - Understand how healthcare, social services, and community resources interplay in managing patient health. - Recognise the emotional and psychological toll of chronic disease on patient well-being.	- Empathy and compassionate observation. - Awareness of patient challenges in accessing care and managing their health. - Insight into patient–healthcare provider communication and relationship dynamics. - Ability to observe and document non-clinical barriers to care.
Reflection and debriefing	- Participation in group discussion sessions led by clinical educators. - Sharing personal observations, focusing on patient’s perspective of navigating healthcare. - Completing structured reflective journals addressing specific prompts. - Discussion on potential solutions and system improvements based on the shadowing experience.	- Deepen empathy through structured reflection. - Cultivate the ability to connect clinical knowledge with patient-centred care insights. - Identify specific social and systemic barriers affecting healthcare delivery. - Generate actionable ideas for improving patient care processes.	- Reflective practice and critical thinking. - Development of problem-solving approaches based on real-world patient experiences. - Interpersonal communication skills and professional empathy. - Ability to apply observations to future clinical decision-making.

**Table 2:** Quantitative results—student evaluations pre- and post-shadowing (summary of numerical improvements in students’ understanding, empathy and confidence, along with comparisons across rotations). Paired *t*-tests showing statistically significant improvements (*p* < 0.001) in all measured outcomes (SDH understanding, empathy, and confidence).

Category	Pre-Programme Results	Post-Programme Results	Details
Understanding of social determinants of health	36% advanced/comprehensive	93% advanced/comprehensive	*p* < 0.001, Cohen’s d = 1.82.
Familiarity with patient-centred care	64% agreed/strongly agreed	90% agreed/strongly agreed	χ^2^(1) = 12.65, *p* < 0.001, η^2^ = 0.23.
Confidence in understanding non-clinical needs	39% agreed/strongly agreed	86% agreed/strongly agreed	Confidence in addressing logistical and emotional patient needs increased significantly (*p* < 0.001).
Empathy score (10-point scale)	6.8 ± 1.2	8.9 ± 1.1	Statistically significant improvement in empathy scores (*p* < 0.001).
SDH Comprehension (Male)	28%	93%	
SDH Comprehension (Female)	59%	91%	
Awareness of logistical barriers	32% aware	82% aware	Awareness of transportation, scheduling, and financial barriers improved substantially.
Understanding of patient health in the context of social factors	35% agreed/strongly agreed	91% agreed/strongly agreed	Significant increase in understanding how housing, family dynamics, and income affect health.
Empathy improvement by rotation (Primary Care)	-	+2.5 points	Students in primary care rotations experienced the largest empathy gains.
Empathy improvement by rotation (Surgery)	-	+2.1 points	Empathy improvements in surgery were linked to observing post-operative recovery challenges.
Empathy improvement by rotation (Medicine)	-	+1.9 points	Medicine rotations showed steady gains, focusing on chronic disease management complexities.
Time spent shadowing (average hours)	-	7.2 h	Students spent an average of 7.2 h observing and engaging with their assigned patients.
Proportion of time in home-based care tasks	-	38%	Observed activities included medication management, dietary planning, and daily routines.
Proportion of time in healthcare appointments	-	62%	Included accompanying patients to GP visits, outpatient clinics, and community services.
Patient interaction frequency (average per day)	-	12 interactions	High interaction frequency provided a diverse understanding of patient challenges.
Awareness of emotional and psychological barriers	45% agreed/strongly agreed	96% agreed/strongly agreed	Recognition of the emotional toll of chronic illnesses increased significantly post-programme.
Preparedness to implement patient-centred approaches	39% agreed	89% agreed	Students expressed readiness to integrate patient-centred care into future practice.
Expectations of learning about patient challenges	86% agreed	-	Pre-programme, students anticipated gaining insight into non-clinical patient experiences.
Overall change in clinical approach	-	89% agreed	Post-shadowing, students reported a commitment to applying programme lessons to patient care.

**Table 3:** Thematic analysis of students’ reflections and feedback (qualitative insights from students on barriers to care, emotional impact, and future practice adaptations).

Theme	Key Insights	Representative Examples	Impact on Students
Barriers to Care	Students consistently observed systemic barriers affecting patients’ ability to manage health.	Transportation issues: “The patient missed appointments because they couldn’t afford reliable transport”.	Increased awareness of how logistical challenges hinder health outcomes.
		Social isolation: “An elderly patient described feeling completely alone in managing their illness”.	Deepened understanding of how isolation exacerbates chronic conditions.
		Polypharmacy: “The patient struggled to manage multiple medications due to confusion over instructions”.	Recognition of the complexity of medication adherence in real-life contexts.
Emotional and Psychological Impact	Students gained insight into the emotional toll of chronic illnesses.	Frustration: “A patient managing COPD expressed how exhausting their daily treatments felt”.	Heightened empathy for the emotional burden patients experience.
		Helplessness: “One patient said they felt like a burden despite trying their best to stay healthy”.	Greater appreciation for the resilience required to navigate systemic barriers.
Daily Health Management	Students observed how patients integrate health management into their daily lives.	“The patient meticulously organised their medications but struggled with inconsistent supply”.	Realised the importance of supporting patients’ self-management efforts.
		“A diabetic patient shared how their dietary restrictions controlled every aspect of their day”.	Gained perspective on the lifestyle challenges associated with chronic diseases.
Healthcare Interactions	Observing appointments highlighted communication and systemic inefficiencies.	“The patient’s GP didn’t address their transport issues, which affected appointment attendance”.	Recognised the need for holistic communication and addressing non-clinical factors in consultations.
		“Community nurses provided vital emotional support, but the coordination with GPs was limited”.	Learned about the value of integrated care and the challenges in achieving it.
Systemic Challenges	Students noted fragmentation between medical and social services.	“The patient lost access to community nursing due to poor coordination between providers”.	Highlighted the need for improved collaboration between healthcare and social support systems.
Role of Families	Family members were key to patients’ health management.	“The patient’s spouse handled all appointments, medications, and doctor communication”.	Understood the critical role families play as caregivers and advocates.
		“A daughter who acted as a carer expressed her own struggles with balancing caregiving and work”.	Gained sensitivity to the needs of caregivers and their dual responsibilities.
Future Clinical Practice	Students reflected on how the shadowing experience would influence their approach to care.	“I now ask patients about their daily routines to understand how treatment fits into their lives”.	Shifted focus to patient-centred approaches that integrate clinical care with social realities.
		“Understanding the patient’s environment showed me why adherence is not just a clinical issue”.	Emphasised holistic care approaches to address social determinants of health.
Proposed Solutions	Students suggested strategies for improving healthcare delivery.	Home-based care: “Regular visits could reduce the burden of transport and improve adherence”.	Advocated for systemic changes to better meet patient needs in their environments.
		Patient navigators: “Having someone help coordinate appointments and medications would make a big difference”.	Recognised the importance of logistical support in improving healthcare accessibility.
		Training: “Doctors should be trained to ask about non-clinical challenges as part of their routine care”.	Stressed the importance of embedding social determinant awareness in medical education.

**Table 4:** Quantitative results—post-session patient feedback (summary of patients’ comfort, understanding, and confidence post-shadowing, specific to surgery patients).

Category	Post-Session Responses (%)	Key Observations
Comfort with having a student shadow	93%	Most patients felt highly comfortable with the presence of students during the shadowing.
Respect for privacy and personal space	93%	Patients noted that students maintained professional boundaries and respected their privacy.
Perception of student interest	100%	Patients observed genuine curiosity and attentiveness from students during their interactions.
Disruption to routine	93%	Patients agreed that students’ involvement did not interfere with their routine care processes.
Programme’s value in educating future doctors	100%	Nearly all patients saw the programme as highly beneficial for improving medical students’ understanding of patient care in clinical settings.
Willingness to recommend programme	91%	Patients were enthusiastic about recommending the programme to others undergoing similar treatments.
Awareness of health resources	79%	Patients reported increased awareness of available aftercare and local health services.
Confidence in self-management	77%	Patients felt more empowered to manage post-operative recovery and symptoms independently.
Clarity of health information provided	89%	Patients found the explanations about their condition and recovery clear and helpful.
Engagement during sessions	91%	Patients actively participated in discussions, appreciating the opportunity to ask questions and clarify doubts.

## Data Availability

The datasets generated and/or analysed during the current quality improvement project are not publicly available due ethical reasons but are available from the corresponding author (W.J.) upon reasonable request.

## Supplementary Materials

The following supporting information can be downloaded at: https://www.mdpi.com/article/10.3390/clinpract15050094/s1, HRA Decision Outcome.

## Appendix A

### Appendix A.1. Student Evaluation Form

#### Appendix A.1.1. Pre-Shadowing Survey

Objective: To assess baseline understanding of patient-centred care and empathy before the shadowing experience.

Instructions: Please rate the following statements based on your current knowledge and expectations.

**Statement**
I understand the concept of social determinants of health and their impact on patient care.
I am familiar with the idea of patient-centred care.
I feel confident in understanding patients’ non-clinical needs (e.g., logistical, emotional).
I understand how factors like transportation and housing affect patient health management.
I believe that empathy plays a critical role in managing patients with chronic conditions.
I expect to learn more about the day-to-day challenges faced by patients living with chronic illnesses.

Open-Ended Questions:

1. What do you hope to gain from this shadowing experience?
2. What do you think will be the most challenging part of shadowing a patient with a chronic condition?
3. How do you currently engage with patients regarding their lives outside of clinical settings?

#### Appendix A.1.2. Post-Shadowing Survey

Objective: To evaluate changes in empathy, understanding, and attitudes toward patient-centred care after the shadowing experience.

Instructions: Please respond to the following statements based on your experience during the shadowing programme.

**Statement**
I now have a better understanding of how social factors (e.g., income, education, family) impact patient health.
My empathy for patients managing chronic conditions has increased as a result of this experience.
I am more aware of the logistical barriers (e.g., transportation, scheduling) that affect healthcare access.
This experience helped me understand the non-clinical challenges that patients face in managing their health.
I now feel more confident in adopting a patient-centred approach in my future clinical practice.
This shadowing experience has changed the way I will approach patient care moving forward.

Open-Ended Questions:

1. What was the most valuable lesson you learned during your shadowing day?
2. How did observing the patient in their daily environment influence your understanding of chronic disease management?
3. What changes do you plan to make in your clinical practice after this experience?

#### Appendix A.1.3. Reflective Journal Prompts

Objective: To encourage comprehensive reflection on the shadowing experience.

Instructions: Use the following prompts to guide your reflective journal, and focus on how this experience has shaped your understanding of chronic disease management.

1. Describe how the patient’s environment and social circumstances affected their health management.
2. Identify the main barriers to care you observed. How did these barriers affect the patient’s adherence to medical treatment or advice?
3. What surprised you most about the patient’s healthcare experience outside the clinical setting?
4. How might this experience change your approach to patient care in the future?
5. Suggest potential improvements or solutions for overcoming the barriers you observed during the shadowing day.

### Appendix A.2. Patient Evaluation Form

#### Post-Shadowing Survey

Objective: To gather feedback from patients about their experience participating in the shadowing programme.

Instructions: Please respond to the following statements by selecting the option that best represents your opinion.

**Statement**
I felt comfortable having a medical student shadow me for the day.
The student respected my privacy and personal space throughout the day.
The student showed a genuine interest in learning about my health and daily challenges.
The student’s presence did not disrupt my usual healthcare routine.
I believe this programme can help future doctors understand the realities of managing chronic illness.
I would recommend this programme to other patients who want to help train future healthcare professionals.

Open-Ended Questions:

1. How did you feel about having a medical student shadow you throughout the day?
2. Was there anything the student did that made you feel uncomfortable? If so, please explain.
3. Do you believe that this programme could improve the way future doctors understand and treat patients with chronic illnesses? Why or why not?
4. Is there any advice you would like to give to future students who participate in this programme?
